# RNA aptamers selected against yeast cells inhibit *Candida albicans* biofilm formation in vitro

**DOI:** 10.1002/mbo3.812

**Published:** 2019-02-18

**Authors:** Boy M. Bachtiar, Chatchawan Srisawat, Endang W. Bachtiar

**Affiliations:** ^1^ Department of Oral Biology Faculty of Dentistry Universitas Indonesia Jakarta Indonesia; ^2^ Oral Research Science Center Faculty of Dentistry Universitas Indonesia Jakarta Indonesia; ^3^ Department of Biochemistry and NANOTEC‐Mahidol University Center of Excellence in Nanotechnology for Cancer Diagnosis and Treatment Faculty of Medicine Siriraj Hospital Mahidol University Bangkoknoi, Bangkok Thailand

**Keywords:** ALISA, aptamer, biofilms, *C. albicans*, SELEX

## Abstract

Aptamers that bind live bacterial cells have been widely investigated, but their potential to inhibit *Candida albicans* biofilm formation needs to be further explored. The aims of this study were to evaluate the binding of *C. albicans* to RNA aptamers and to examine the potential of aptamers to inhibit *C. albicans* biofilm formation in vitro. In this study, RNA aptamers selected against yeast cells of *C. albicans* ATCC 10231 were developed using the systematic evolution of ligands by exponential enrichment (SELEX) technique. The binding affinity of the resulting aptamers was then determined by an aptamer‐linked immobilized sorbent assay (ALISA), and a colorimetric (MTT) assay was used to measure the metabolic activity of *Candida* biofilms. After 11 rounds of SELEX, two candidate aptamers, Ca‐apt‐1 and Ca‐apt‐12, were identified. The Ca‐apt‐1 aptamer also recognized *C. albicans* isolated from clinical specimens but did not recognize other oral microorganisms (i.e., *Streptococcus mutans* and *Saccharomyces cerevisiae*). The ALISA results showed that the binding affinity of these aptamers was comparable to that of an anti‐*C. albicans* monoclonal antibody. In addition, Ca‐apt‐1 could inhibit biofilm and hyphal formation of *C. albicans* in vitro*,* as demonstrated using biofilm assays. This study shows that RNA aptamers could potentially be used in diagnostic and therapeutic applications for *C. albicans*‐related disease in the future.

## 1 INTRODUCTION

*Candida albicans* is a component of normal human flora and an opportunistic pathogen (Akpan & Morgan, 2002; Soll, 2002). Compared to other fungal pathogens that exist primarily in either yeast or hyphal forms, *C. albicans* shows phenotypic plasticity because it has the ability to switch between different morphological forms in response to environmental cues (Whiteway & Bachewich, 2007). This morphogenic switching from yeast to hyphal form contributes to the overall virulence of *C. albicans* (Bastidas, Heitman, & Cardenas, 2009). Because of this characteristic associated with its virulent character, developing antifungals that target the early phase of *C. albicans* biofilm formation is challenging.

Aptamers are short, single‐stranded oligonucleotides that have emerged as a new class of small molecule ligands that can recognize and bind specific target molecules with high affinity and specificity (Jayasena, 1999). Aptamers bind target molecules with the affinity and specificity equal to or greater than those of antibodies (Tang et al., 2007). Aptamers are selected by an in vitro selection process called SELEX (systematic evolution of ligands by exponential enrichment) (Tuerk & MacDougal‐Waugh, 1993). Although numerous reports have detailed the selection of aptamers against different bacterial species (Cao et al., 2009; Chen, Zhou, Luo, Mohammed, & Zhang, 2007; Hamula, Le, & Li, 2011), few studies screening aptamers for their clinical value and potential use to inhibit the morphology switching that occurs during *C. albicans*‐related infections have been reported.

In this study, we used the SELEX technique to select RNA aptamers with high affinity and specificity for *C. albicans* yeast cells. Furthermore, we used an aptamer‐linked immobilized sorbent assay (ALISA) to demonstrate the potential use of high‐affinity aptamers in quantitative determination of *C. albicans*. Finally, we investigated the ability of the aptamers to inhibit *C. albicans* growth in vitro.

## 2 MATERIALS AND METHODS

### 2.1 Preparation of cells for aptamer selection

*Candida albicans* (ATCC 10231) was used as a targeted aptamer ligand, while *Saccharomyces cerevisiae* (ATCC 9763) was used for counter selection. For the binding specificity test, we used *Streptococcus mutans* (Xc), a stock culture of *C. albicans* that was previously isolated in our dental hospital (Universitas Indonesia) using Chromogenic Candida Agar (CCA; Oxoid, Basingstoke, UK) (Ghelardi et al., 2008), and *S. cerevisiae*. All microorganisms were maintained and propagated as described elsewhere (Bachtiar et al., 2014; Shibata, Kawada, Nakano, Toyoshima, & Yamashita, 2005; Vazquez‐Reyna, Balcazar‐Orozco, & Flores‐Carreon, 1993). To obtain budding yeast cells of *C. albicans* and *S. cerevisiae*, we used YPD (1% yeast extract, 2% peptone, 2% glucose) broth shaking at 30°C that was inoculated with *C. albicans* or *S. cerevisiae* that had been grown overnight on a YPD agar plate under aerobic conditions. The resulting budding yeasts were washed twice in phosphate‐buffered saline (PBS, Oxoid Ltd, Basingstoke, UK) and resuspended in RPMI 1640 supplemented with L‐glutamine and buffered with MOPS (Sigma, St Louis, MO, USA). The yeast density was measured by using a hemocytometer and adjusted for the SELEX procedure, while the bacterial number was counted by the plating method.

### 2.2 In vitro selection of RNA aptamers for *C. albicans* ATCC 10231

A library of RNAs containing 40‐nt randomized central sequences flanked by defined primer binding sites with the sequence of 5′‐GGGAGUCGACCGACCAGAA [N40] UAUGUGCGUCUACAUCUAGACUCAU‐3′ (84 nt) was generated as previously described (Srisawat & Engelke, 2001) with a calculated library complexity of 1 × 10^13^ different RNA sequences. The selection conditions of the SELEX process are shown in Table 1. The specified amounts of RNA and yeast were mixed in a 0.45‐μm spin column (Millipore), and the binding reaction was performed in a total volume of 50 μl in the binding buffer (50 mmol/L HEPES pH 7.4, 10 mmol/L MgCl_2_, 100 mmol/L NaCl) with 5 μg of baker's yeast tRNA. The reaction was incubated at room temperature for 45 min in rounds 1–5 and for 30 min from round 6 onwards with gentle rotation. The cells were then washed with the binding buffer, and the bound RNAs were eluted with 500 μl of the elution buffer (8 mol/L urea, 5 mmol/L EDTA, pH 8.0). The eluted RNAs were then recovered by ethanol precipitation, amplified by quantitative real time‐PCR (q‐PCR), and transcribed in vitro to generate RNAs for the next round of selection (Srisawat & Engelke, 2001). To enrich the aptamers specific to *C. albicans*, a counterselection step was included at rounds 3, 5, and 10 using 5 × 10^7^ *S. cerevisiae* cells. The RNAs unbound after *S. cerevisiae* binding were used in the binding reaction with *C. albicans* as described above.

**Table 1:** *Candida albicans*‐specific aptamer selection protocol*

Round	Input RNA (pmol)	*C. albicans* (cells)	Washing
1	100	5 × 10^9^	100 μl 5 times, 1 min each
2	25	5 × 10^8^	100 μl 5 times, 3 min each
3	25	5 × 10^8^	100 μl 5 times, 3 min each
4	12.5	5 × 10^7^	100 μl 5 times, 5 min each
5	12.5	5 × 10^7^	100 μl 5 times, 5 min each
6	6.25	5 × 10^6^	100 μl 5 times, 10 min each
7	6.25	5 × 10^6^	100 μl 5 times, 10 min each
8	3.13	5 × 10^5^	100 μl 5 times, 15 min each
9	3.13	5 × 10^5^	100 μl 5 times, 15 min each
10	1.56	5 × 10^4^	100 μl 5 times, 15 min each
11	1.56	5 × 10^4^	100 μl 5 times, 20 min each

* *Saccharomyces cerevisiae* was used in a subtraction step at rounds 3, 5, and 10.

After 11 rounds of selection, the RNAs were cloned into a plasmid using a TOPO TA Cloning Kit, and the ligated plasmids were transformed into One Shot^®^ Top 10 *Escherichia coli* (Invitrogen, Carlsbad, CA). The plasmids containing the aptamers were purified using a QIAprep Miniprep Kit (Qiagen, Hilden, Germany), and the aptamer sequences were determined by First BASE Laboratories Sdn Bhd (Malaysia). The obtained RNA sequences were further evaluated for binding affinity and specificity as follows: approximately 10^6^ tested cells were mixed with 100 pmol of either the control or *C. albicans*‐specific aptamers in the presence of baker's yeast tRNA. After the binding reaction, washing, and elution steps described above, the amount of input and bound RNAs were quantified using q‐PCR, and the binding percentage was calculated as bound RNAs × 100/input RNAs. Predicted secondary structures were generated using RNAstructure 5.3 (Reuter & Mathews, 2010).

### 2.3 Aptamer‐linked immobilized sorbent assay

The binding affinity of the selected aptamers was measured using an ALISA. To do this, 96‐well microtiter plates (Iwaki, Tokyo, Japan) were coated with 50 μl of mouse anti‐human *C. albicans* monoclonal antibody (U.S. Biological, Swampscott, Mass) diluted to 1 μg/ml in coating buffer (50 mmol/L Na_2_CO_3_, pH 9.6) and incubated overnight at 4°C. The plates were washed twice with PBST (50 mmol/L phosphate‐buffered saline, pH 7.2 containing 0.05% Tween 20) and blocked with 100 ml of 1% BSA (Sigma, MA, USA) in PBST for 90 min at room temperature (RT). After washing, various concentrations of the *C. albicans* preparation in PBST (100 μl, triplicate), ranging from 50 to 5,000 cells/ml, were incubated for 1 hr at RT by gentle shaking in 100 μl of binding buffer. After the designated time, the unbound target was removed, and the plates were washed twice with PBST containing 0.1% Tween 20. Subsequently, 100 μl (100 μg/ml) of the biotinylated aptamer that was prepared as described elsewhere (Tsuji et al., 2009) was added into each well, and binding was allowed to proceed protected from light for 1 hr at RT.

The unbound materials were removed by washing with washing buffer (three times). Finally, 100 μl of a 1:1,000 dilution of a solution of streptavidin conjugated to horseradish peroxidase (HRP) was added to the individual wells. Following a 30‐min incubation on a shaking platform at RT, wells were washed twice with PBST and developed using ABTS as a substrate (Sigma, MA, USA). The reaction was stopped with 100 ml of 0.25 M H_2_SO_4_, absorbance was measured at 450 nm using a microplate reader (BioRad, USA), and washing buffer was used as a background control. The same procedure was used for enzyme‐linked immunosorbent assays (ELISA). However, the biotinylated aptamer used in the ALISA was replaced by biotinylated polyclonal anti‐*C. albicans* antibodies in the ELISA.

### 2.4 The effect of RNA aptamers on biofilm formation

Biofilm formation assays were performed as previously described (Bachtiar et al., 2014). Briefly, 100 μl containing 1.8 × 10^5^ yeast cells of *C. albicans* from overnight culture at 35°C was aliquoted into microtiter plates. Three different concentrations of each tested aptamer in buffer (1 ng/μl, 10 ng/μl, and 10 ng/μl) were then added into separate wells, and the plates were incubated at 37°C in 5% CO_2_ in air for 90 min with gentle shaking. To promote biofilm formation, the wells were treated with 150 μl of fresh yeast nitrogen base (Sigma‐Aldrich) medium without aptamer, and the culture period was lengthened to 24 hr. *Candida albicans* growth was determined by evaluating the metabolic activity of growing *C. albicans* using MTT (3‐(4,5‐dimethylthiazol‐2‐yl)‐2,5‐diphenyltetrazolium bromide) reagent (Sigma‐Aldrich). *Candida albicans* biofilms with PBS (pH 7.2) added instead of aptamer were included to serve as negative controls. Statistical significance for all experiments was determined by pairwise comparison by *t* test using GraphPad Prism 7.0 software. *p* < 0.05 was considered statistically significant.

## 3 RESULTS

### 3.1 In vitro selection of RNA aptamers against yeast cells of *C. albicans*

In this study, q‐PCR was used to monitor the enrichment progress of RNA aptamers. To decrease nonspecific binding of RNA, the experimental conditions in each selection round (Table 1) included 5 μg of baker's yeast tRNA as a binding competitor in a volume of 50 μl. The obtained RNA pool was further utilized to select for aptamers that specifically bind to live *C. albicans* yeast cells.

**Figure 1:**
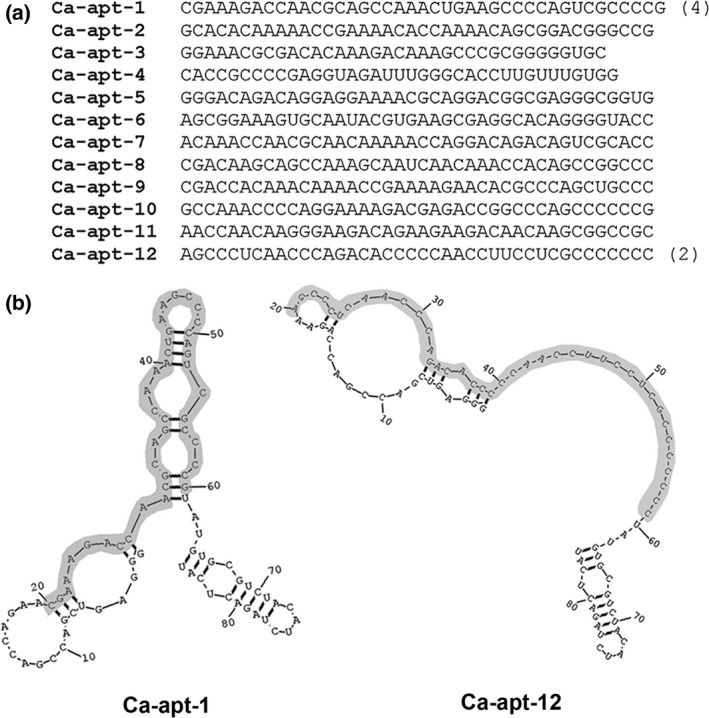
The sequences and predicted secondary structure of the *Candida albicans*‐specific aptamers (Ca‐apt). (a) The randomized region of the aptamers is shown. The number in parentheses represents the number of clones identified during the aptamer screening. (b) The predicted secondary structures of Ca‐apt‐1 and Ca‐apt‐12 with the lowest folding energy are shown. The nucleotides in the shaded area correspond to the randomized region of the aptamer

From the first to fifth rounds of the SELEX process, the components of the library that were enriched included specific and nonspecific binders. However, when the counterselection step was performed at the fifth round, the retention rate decreased gradually from the sixth to 10th rounds. We found that at the 11th round, the enrichment of the RNAs capable of binding to the target *C. albicans* increased significantly compared with the fifth round RNAs and the original library RNAs (data not shown). Therefore, the PCR products from round 11 were cloned to characterize each aptamer, and the randomized regions of the aptamers are shown in Figure 1a. They do not exhibit any consensus sequences and are unique except for Ca‐apt‐1 and Ca‐apt‐12, which were identified four and two times during the screening, respectively. Their preferential retention during selection suggests that they might have high affinity toward the target cells. The predicted secondary structures of both aptamers are shown in Figure 1b. Interestingly, the length of the randomized region of Ca‐apt‐1 [41 nucleotide (nt)] was longer than that of the original 40‐nt pool RNAs. Such changes may occur either during reverse transcription or PCR steps in multiple rounds of SELEX (Doudna, Cech, & Sullenger, 1995; Takemura et al., 2006; Ye et al., 2014).

**Figure 2:**
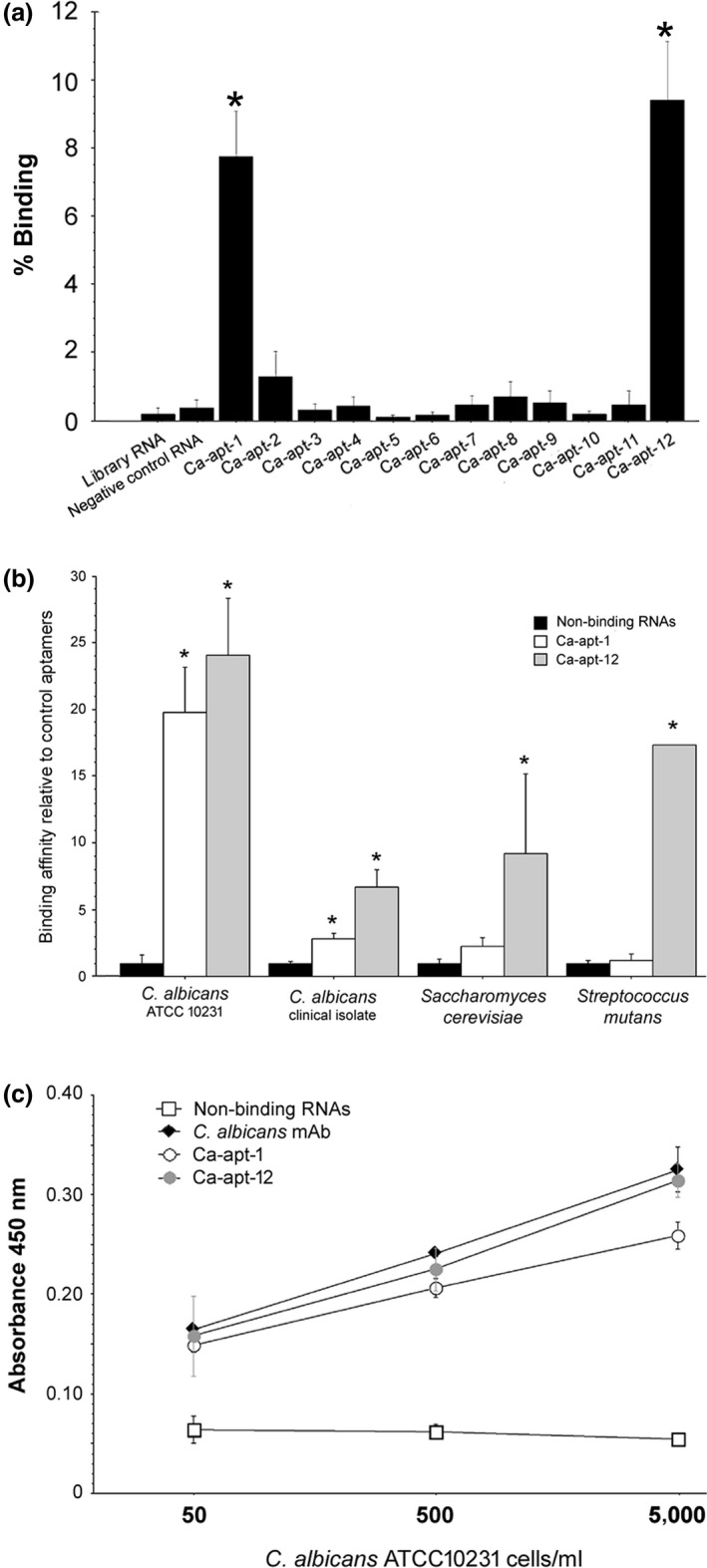
The binding properties of the *Candida albicans*‐specific aptamers. (a) The aptamers were screened for their binding to the *C. albicans* ATCC 10231 strain, which was used as the selection target. The plot shows the binding percentage (bound RNA × 100/input RNA). Only Ca‐apt‐1 and Ca‐apt‐12 demonstrated significantly higher binding than the negative control RNA. (b) The specificity of Ca‐apt‐1 and Ca‐apt‐12 was tested using either the target *C. albicans* strain or a clinical strain isolated from the oral cavity, a related yeast strain *Saccharomyces cerevisiae*, and *Streptococcus mutans*. The aptamer‐linked immobilized sorbent assay (ALISA) using both aptamers is shown and compared with an ELISA using antibodies against *C. albicans*. The plots show the mean values, and an error bar represents the standard error of the mean (*SEM*). An asterisk indicates a statistically significant difference compared with the nonbinding RNAs using an unpaired *t* test (*p* < 0.05)

Next, the aptamers were screened for their binding to the target *C. albicans*. As expected, only the Ca‐apt‐1 and Ca‐apt‐12 aptamers demonstrated significantly higher binding percentages than the negative control RNA, which is a clone randomly chosen from the original RNA library (Figure 2a). Therefore, these aptamers were chosen for subsequent characterization.

### 3.2 Binding specificity test

To test the specificity of the aptamers and their binding to various targets, that is, *C. albicans* strains, either from the ATCC or a clinical specimen, *S. cerevisiae*, and *S. mutans*, was evaluated. As shown in Figure 2b, Ca‐apt‐1 and Ca‐apt‐12 aptamers show significantly higher binding affinities to *C. albicans* ATCC strain compared with those of the nonbinding RNAs, which are the aptamer clones showing no target binding. Moreover, both aptamers can also recognize *C. albicans* from a clinical specimen albeit at somewhat lower binding percentages (Figure 2b).

To confirm the results and to test whether the aptamers can be used in diagnostics for *C. albicans*, we evaluated the aptamers ability to detect *C. albicans* using the ALISA method. The results showed that both Ca‐apt‐1 and Ca‐apt‐12 can detect *C. albicans* at concentrations ranging from 50 to 5,000 cells/ml, and the ALISA performance is comparable to that of the ELISA method using *C. albicans*‐specific antibodies (Figure 2c).

### 3.3 Biofilm inhibition assay

**Figure 3:**
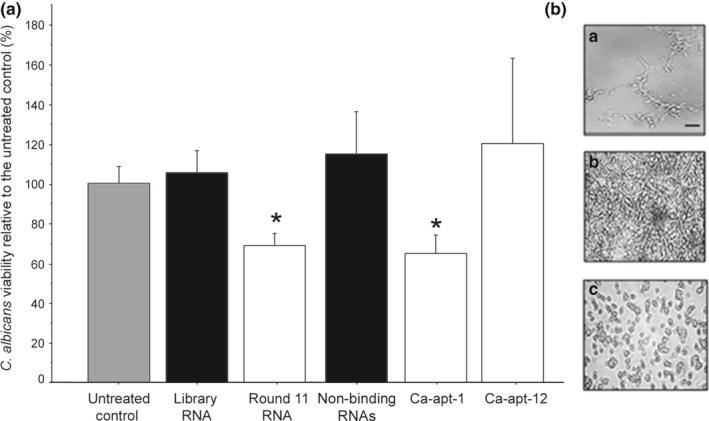
The effect of the *Candida albicans*‐specific aptamers on *C. albicans* biofilm formation. (a) *C. albicans* viability was determined after 24 hr of growth using the MTT assay in the presence of the RNAs from the original library, round 11, nonbinding RNAs, Ca‐apt‐1, and Ca‐apt‐12 at a concentration of 1 ng/μl. The plots show the mean values of the relative viability compared to the untreated control, and an error bar represents the standard error of the mean. An asterisk indicates a statistically significant difference compared with the untreated control using an unpaired *t* test (*p* < 0.05). (b) Representative light microscopy images of *C. albicans* in the untreated control (top) or the cells treated with either the original library RNA (middle) or Ca‐apt‐1 (bottom) are shown. The images from the light microscope (400× magnification) were edited for brightness and contrast, and the bars represent 20 μm for all images. Decreased hyphal formation is observed in the presence of the aptamer

The RNAs from the original library, round 11 RNAs, nonbinding RNAs, Ca‐apt‐1, and Ca‐apt‐12 were preincubated with *C. albicans* yeast cells before the biofilm was allowed to form. As shown in Figure 3a, the MTT assay results after 24 hr of biofilm growth demonstrate that the presence of the round 11 RNA and the Ca‐apt‐1 aptamer can cause a significant reduction in cell viability compared with that of the untreated group (*p* < 0.05). Moreover, there was a decrease in hyphal formation from yeast cells treated with the Ca‐apt‐1 aptamer compared with the untreated or library RNA‐treated group, as observed under a light microscope (Figure 3b). Interestingly, the Ca‐apt‐12 aptamer did not seem to affect biofilm formation, although it demonstrates binding affinity to *C. albicans*, as shown by ALISA (Figure 2c). Moreover, incubation of *C. albicans* during the early stage of biofilm formation with Ca‐apt‐1 at concentrations as low as 1 ng/μl was sufficient to inhibit viability and hyphal formation.

## 4 DISCUSSION

*Candida albicans* is the most common fungus causing oral candidiasis and is involved in the pathogenesis of early childhood caries (Falsetta et al., 2014). However, no vaccine against this oral opportunistic pathogen is currently available. Thus, effective strategies for detecting and inhibiting *Candida* infection are needed. Aptamers have been reviewed in detail as highly sensitive and specific ligands to detect pathogens (Torres‐Chavolla & Alocilja, 2009). In this study, whole yeast cells of *C. albicans* strain ATCC 10231 were used as the target of RNA aptamer selection, as this may generate multiple targets in parallel (Shangguan et al., 2008) toward *C. albicans* cell surface molecules. Using whole‐cell targets in the SELEX process can be faster, easier, and more reproducible than using other targets (Guo, Lin, Zhang, Simon, & Kushner, 2009). In addition, when using the whole‐cell SELEX method, it is not necessary to isolate and purify a single target protein, which might change the presentation of the target molecule compared to an intact, live, bacterial cell (Dwivedi, Smiley, & Jaykus, 2010). Thus, this study showed that using the whole cell as a targeted ligand in the SELEX procedure has the potential to result in aptamers with binding affinity for targets on the cell in their native conformations.

In the current study, we used q‐PCR to monitor library enrichment after each selection cycle, showing relatively increasing amounts of RNA binding that reached a maximum in the 11th round. To increase aptamer binding, we used a relatively low number of *C. albicans* cells*,* as studies have shown that compared with using a large amount of target, using small amounts of target increases the success of the selection and often results in higher‐affinity aptamers (Chen et al., 2007).

When the specificities of our samples were compared, our data showed that the Ca‐apt‐1 aptamer bound to only *C. albicans*, whereas the Ca‐apt‐12 aptamer can also bind to the related yeast species *S. cerevisiae* and the oral bacterium *S. mutans*, which has a symbiotic relationship with *C. albicans* (Falsetta et al., 2014). As this study cannot determine the target molecule of the aptamer, we speculated that some of the potential targets of Ca‐apt‐12 are mannoproteins, immunodominant outer cell wall components of *C. albicans* (Lopez‐Ribot, Casanova, Murgui, & Martinez, 2004). These glycoproteins mediate *C. albicans*–*S. mutans* interplay in plaque biofilm (Bachtiar & Bachtiar, 2018; Hwang et al., 2017), and they have a critical role in the pathogenesis of early childhood caries (Kim et al., 2017). However, additional studies are required to identify the possible targets of both aptamers.

The aptamers were further tested for their effects on *C. albicans* biofilm formation, which is considered to contribute to the virulence characteristics of *C. albicans* (Chandra et al., 2001).We found that the Ca‐apt‐1 has the ability to interfere with *C. albicans* biofilm formation at the intermediate stage (24 hr) (Bachtiar, Dewiyani, Akbar, & Bachtiar, 2016) because the aptamer must act at the earliest stage of biofilm formation, which was set at 90 min in our experiment. We hypothesized that the effects of the Ca‐apt‐1 aptamer on biofilm formation might be physicochemical in nature or due to direct contact between the aptamer and the fungus, as shown in this study by a binding affinity test using an ALISA. However, in this study, we cannot explain the mechanisms by which the aptamer interferes with biofilm formation. Thus, further studies are necessary.

In conclusion, in this study, two candidate RNA aptamers, Ca‐apt‐1 and Ca‐apt‐12, were obtained through the SELEX method. The Ca‐apt‐1 aptamer binds specifically to *C. albicans* and possesses the ability to inhibit the fungus as it develops as a biofilm, whereas Ca‐apt‐12 shows cross‐binding with *S. cerevisiae* and *S. mutans* and does not affect biofilm formation. Additional studies are necessary to identify the aptamer targets and to explore the potential applications of these aptamers in clinical settings.

## CONFLICT OF INTEREST

The author(s) declare no financial or commercial conflicts of interest.

## AUTHORS CONTRIBUTION

BMB and CS designed, coordinated, and performed the experiments, as well as drafted the manuscript. EWB and CS analysed the data. All authors read and approved the manuscript.

## ETHICS STATEMENT

None declared.

## Data Availability

All data are included in the manuscript. Raw data are available on request.
